# Jet Mode Recognition of Electrohydrodynamic Direct-Writing Based on Micro/Nano Current

**DOI:** 10.3390/mi11020128

**Published:** 2020-01-23

**Authors:** Guoyi Kang, Gaofeng Zheng, Yanping Chen, Jiaxin Jiang, Huatan Chen, Xiang Wang, Wenwang Li, Yuqing Huang, Jianyi Zheng

**Affiliations:** 1 Department of Instrumental and Electrical Engineering, Xiamen University, Xiamen 361102, China; 35120190154070@stu.xmu.edu.cn (G.K.); ypchen@xmu.edu.cn (Y.C.); jiangjx@xmu.edu.cn (J.J.); 35120190154074@stu.xmu.edu.cn (H.C.); 2 Shenzhen Research Institute of Xiamen University, Shenzhen 518000, China; 3 School of Mechanical and Automotive Engineering, Xiamen University of Technology, Xiamen 361024, China; wx@xmut.edu.cn (X.W.); xmlww@xmut.edu.cn (W.L.); 4 Department of Electronics Science, Xiamen University, Xiamen 361005, China; yqhuangw@xmu.edu.cn

**Keywords:** electrohydrodynamic direct-writing, micro/nano current detection, jet mode recognition, current characteristics, Kaiser filter

## Abstract

The online recognition of jet mode is important for the accurate control and further application of electrohydrodynamic direct-writing (EDW) technology. An EDW system with a current detection module is built for jet mode recognition. The current of the EDW jet is measured to recognize the jet mode when printing patterned structures. Then, a data processing program with a digital Kaiser low-pass filter is developed in MATLAB, via which the noise of the current signal is reduced. The features of EDW current, including the current fluctuation and the peak current intervals, are studied to recognize different jet modes. The current characteristics of three jet modes are investigated: droplet ejection mode, Taylor cone ejection mode, and retractive ejection mode. The Taylor cone ejection mode has the smallest coefficient of variation of peak current. This work provides a good way of designing the optimized control algorithm and of realizing the closed-loop control system, which contributes to enhancing the jet stability and accelerating the application of EDW technology.

## 1. Introduction

With the advantages of low cost, simple process, easy integration, and good material compatibility, electrohydrodynamic direct-writing (EDW) technology has great potential in flexible electronics [1,2], biological tissue engineering [3,4] cell engineering [5,6], and other fields. Based on the electrohydrodynamic (EHD) theory, the electrostatic voltage is loaded on the nozzle tip to stretch the solution into a fine jet for the printing of micro/nano structures, including nanofibers, droplets, and bead-on-string structures [7,8,9,10], which can be operated under room temperature and normal pressure conditions [11]. By shortening the distance between the nozzle and the collector, the stable and straight jet can be used to direct-write precise micro/nano patterns [12,13]. The accurate deposition control of the charged jet is the key for the application of EDW technology [14].

There are several ejection modes for EHD printing, including the micro-dripping mode, spindle mode, cone-jet mode, and multi-jet mode [15]. The continuous fiber with high accuracy is a typical structure for precise micro/nano additive manufacturing, which can only be obtained using the cone-jet mode. The fine jet is generated from the tip of Taylor cone, and the diameter is independent of the inner diameter of the nozzle to decrease the characteristic dimension of printed structures. On the other hand, the fine jet is constrained at the tip of the Taylor cone by the electrical field, which can be used for the precise deposition of micro/nano structures. Due to the short ejection distance and motion time, various ejection modes lead to different structure features and deposition accuracy of printed patterns, while also influencing the size and uniformity of printed structures. Thus, the recognition and control of the jet mode are necessary for the application of EDW in integration manufacturing of micro/nano systems.

During the EHD printing process, the charges are transferred along the jet from the nozzle to the ground through the collector, forming a micro/nano current, which is an important parameter when investigating the identification and stability control of the charged jet [16,17]. Bhattacharjee et al. [18] studied the theory of electrospinning current and found that the current contained two parts, the ohmic current formed by the charge transport and the leakage current formed by the high voltage. Munir et al. [19] designed a current detection device to realize the real-time monitoring of the electrospinning current, via which the characteristics of current and nanofiber diameter in different jet modes were studied.

The current characteristics can reflect jet behaviors of EDW technology. Bober et al. [20] studied the unstable motion behavior and the control mechanism of EDW under different parameters. Wang et al. [21] clarified the relationship between the EDW current and the jet mode, which proved that the EDW current could effectively reflect the Taylor cone state. Different jet ejection modes lead to various micro/nano structures [22]; thus, the online recognition of jet mode is important for the precise closed-loop control of EDW technology. The EDW current is a direct parameter reflecting the jet ejection behavior, which can be used to help recognize the jet modes. However, due to the short nozzle-substrate distance, fine jet, and multi-parameter interferences, the EDW current is only several nanoamperes, featuring a small amplitude, large fluctuation range, large background noise, and fast response speed. Therefore, the accurate detection and evaluation of the current quantitative feature when printing patterned structures is the key to jet mode recognition for accurate deposition of micro/nano patterns.

This paper focuses on the accurate recognition of the jet mode based on the EDW current when printing micro/nano patterns. An accuracy current detection system is established to investigate the EDW current characteristics under different jet modes during the direct-writing of patterned structures. The EDW current signals are filtered to obtain the quantitative feature characteristics and identify different jet modes accurately. The relationship of the current characteristics and the ejection modes is investigated, which can be the basis of the closed-loop control system of EDW.

## 2. Materials and Methods

### 2.1. Detection and Measurement System of Micro/Nano Current

**Figure 1:**
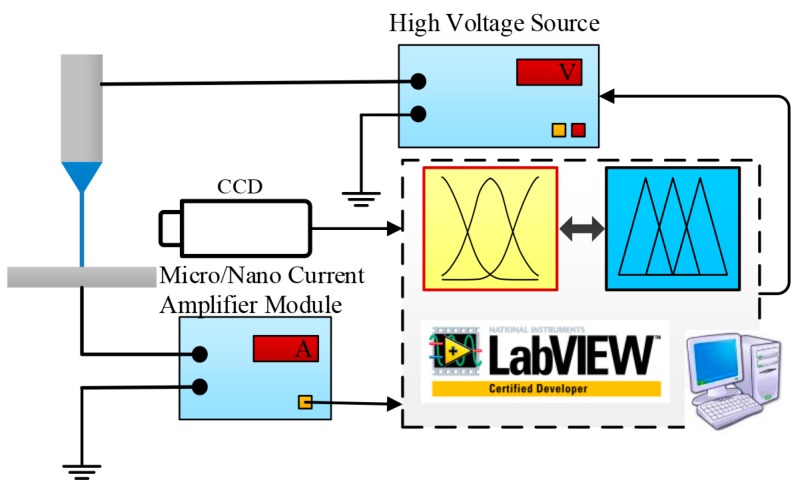
The current detection and measurement system of electrohydrodynamic direct-writing (EDW).

An experimental system with a micro/nano current detection module was built, as shown in Figure 1, including a precision syringe pump (Pump 11 Pico Plus Elite, Harvard Apparatus America, Holliston, MA, USA), a high-voltage power supply (DW-SA403-1ACE5, Dongwen High Voltage Power Source Ltd., Tianjin, China), a spinneret (inner diameter 60 μm), a motion platform, a collector, a charge-coupled device (CCD) camera (UI-2250-C, IDS Imaging Development Systems GmbH, Obersulm, German), a micro/nano current detection module, and a host computer. The host computer and the high-voltage power supply were connected via RS232 communication through the Universal Serial Bus (USB) port. The anode of the high-voltage power supply was connected to the nozzle. The precision syringe pump was used to supply solution to the nozzle. The grounded copper collector was fixed in the shielding box of the micro/nano current detection module and placed directly under the nozzle. The output of the micro/nano current detection module was connected to the data acquisition card, via which the current data were delivered to the host computer. The CCD camera was connected to the host computer through the USB port to observe the jet ejection behavior.

The micro/nano current monitoring system consists of an amplifying circuit and a data acquisition module. The EDW current ranges from tens of nanoamperes to a few microamperes [23]. However, due to the electrical field interferences and random factors, the strong noise interferences are a great obstacle for the accurate measurement of EDW current. In our work, the current-voltage conversion amplification method was adopted in the amplifier module. The current signal was converted into a voltage signal, and the conversion resistor was connected to the inverting output of the amplifier module, showing advantages of high sensitivity and simple structure to reduce the interferences [24].

### 2.2. Micro/Nano Current in Printing Patterns

**Figure 2:**
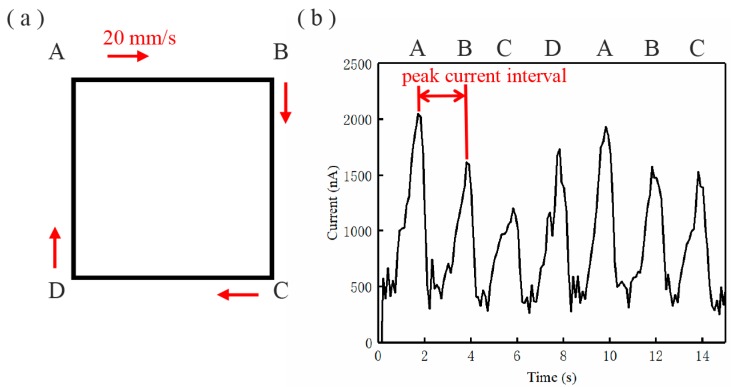
Micro/nano current in printing patterns: (**a**) motion trajectory of collector; (**b**) corresponding current waveform (applied voltage of 2 kV).

The motion trajectory of the collector in Figure 2a was designed to investigate the characteristics of micro/nano current. The collector moved in a square pattern with a side length of 10 mm at the velocity of 20 mm/s. Then, 10 wt% polyethylene oxide (PEO, molecular weight (*M*_w_) = 300,000 g/mol) solution was used as the EDW material, of which the PEO powder was dissolved in the mixture solvent of deionized water and absolute ethanol (*v*:*v* = 3:1). The experiments were conducted at room temperature between 20 °C and 24 °C and relative humidity between 40% and 60%. The distance between the spinneret and the collector was 4 mm. The liquid supply rate was 20 μL/h. The moving collector provided a mechanical stretching effect on the viscous jet, and the jet deviated from the vertical line under the stretching force from the collector, making it difficult to obtain precise micro/nano patterns [25,26]; thus, the collector stopped at each turning point for 1.5 s. When the collector stopped, the jet whipped at the turning point position of the pattern, and more charges deposited on the collector in a short time, increasing the electrical interferences and resulting in the current peaks shown in Figure 2b. The peak current interval is an important value to characterize the repeatability of EDW current when printing patterned structures.

### 2.3. Data Processing of Micro/Nano Current

**Figure 3:**
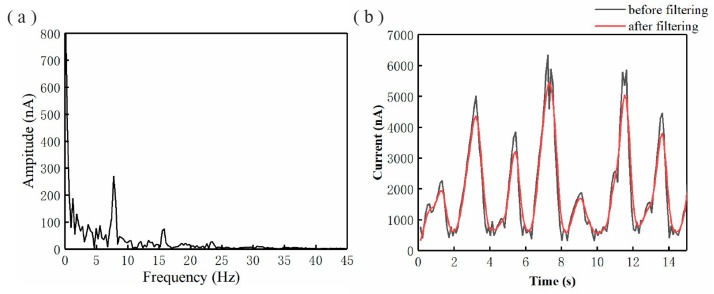
Data processing of micro/nano current: (**a**) amplitude spectrum of EDW current signal; (**b**) EDW current before and after filtering.

The EDW current data from the current detection module were collated and stored in an Excel spreadsheet through the host LabVIEW program (LabVIEW 2014, National Instruments, Austin, TX, USA). Then, a MATLAB program (MATLAB 2014, MathWorks, Natick, MA, USA) was prepared to directly read the data from the data sheet. Due to the high voltage and strong interferences, there were several noise signals in the weak EDW current, making it difficult to achieve jet mode recognition. Fast Fourier transform (FFT) was performed by the MATLAB program to gain the current amplitude spectrum, as shown in Figure 3a. The micro/nano current signal mainly comprised frequencies below 10 Hz, in line with experimental expectations without 50 Hz power frequency noise. The current signal had a small amplitude, but it was relatively evenly distributed at various frequencies larger than 20 Hz. A data processing program with a digital low-pass Kaiser filter was built to reduce the noise interference, for which the passband cutoff frequency was set to 10 Hz, the stopband cutoff frequency was set to 14 Hz and the stopband minimum attenuation was set to −80 dB. The current was measured when the applied voltage was 2.5 kV. The current waveforms before and after filtering are shown in Figure 3b. The low-pass filter reduced the noise and smoothed the current waveform, making it easier to gain the characteristics of micro/nano current.

## 3. Results and Discussions

With the increase in applied voltage on the metal nozzle, four jet ejection modes occurred sequentially [27]: droplet ejection mode, Taylor cone ejection mode, retractive ejection mode, and forked ejection mode. The forked ejection mode, which should be avoided during the EDW process, can be recognized easily using simple image processing methods. However, for the other three stable jet ejection modes, feature identification is important for the accurate control of printing precise patterns.

**Figure 4:**
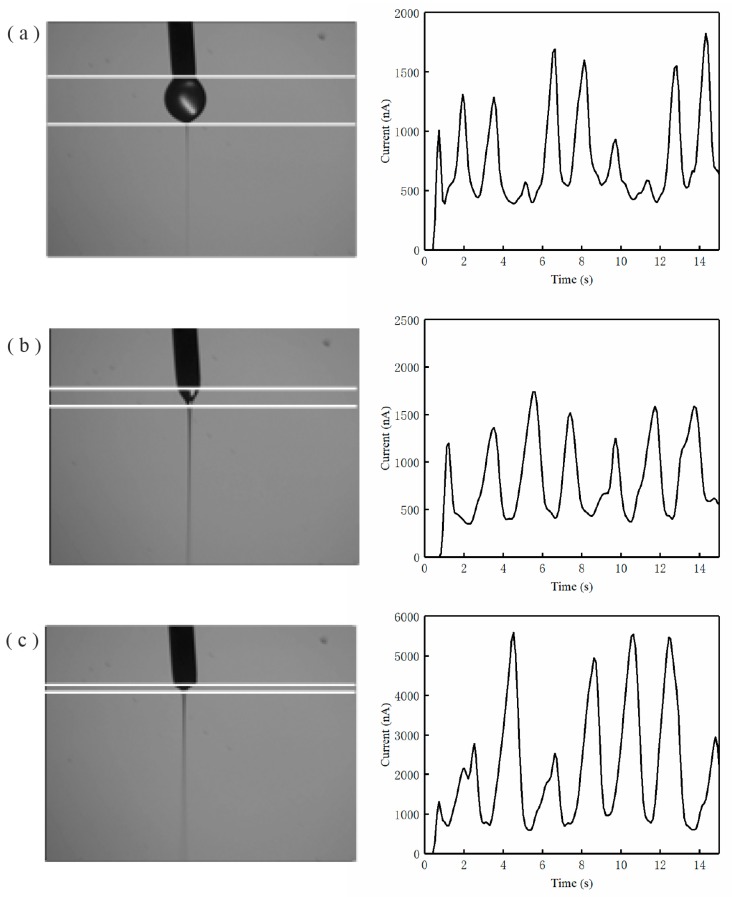
Jet mode recognition with current waveform: (**a**) droplet ejection mode; (**b**) Taylor cone ejection mode; (**c**) retractive ejection mode.

The EDW micro/nano currents under different jet modes are shown in Figure 4, in which the jet images were taken at the turning point position. There was a current peak at each turning point of the square trajectory. The peak current intervals depicted in Figure 2b for each jet mode were 2 ± 0.1 s, corresponding to the moving trajectory of the collector, indicating that the EDW current could reflect the jet behavior in printing patterns. Using the data processing program, the noise signal was reduced and there were independent current peaks at the turning point position of the trajectory for each jet mode. It can be seen that the Taylor cone ejection mode showed the most stable repeatability during the EDW process in printing each side of the pattern.

The feature characteristics of micro/nano current under different jet modes are illustrated in Table 1, which can be used to recognize the jet modes. *I*_min_ and *I*_max_ are the minimum and maximum current in each period. *I*_mean_ is the mean of the current during the printing process. *CV* is the coefficient of variation of peak current. Twenty experimental trials are performed for each mode. A *CV* of 0.5 was taken as a boundary value. If the *CV* was less than 0.5, the EDW jet was identified to be in the Taylor cone ejection mode. The *CV* of the droplet ejection mode and retractive ejection mode was relatively close. The applied voltage in the retractive ejection mode was larger than that in the droplet ejection mode; thus, more charges gathered at the turning point position of the trajectory, leading to a sharper peak in the current waveform. Therefore, these two modes could be identified according to the ratio of *I*_max_ to *I*_min_. If the *CV* was higher than 0.5, when the ratio was between 4 and 7, the EDW jet was identified to be in the droplet ejection mode. On the other hand, when the ratio was larger than 7, the EDW jet was identified to be in the retractive ejection mode. In this way, the accurate recognition of jet mode can be realized using the feature characteristics of micro/nano current, providing effective closed-loop control of EDW technology.

## 4. Conclusions

Focused on the recognition of EDW jet mode, a micro/nano current detection system and a data processing program with a Kaiser low-pass filter were designed to obtain the characteristics of the EDW current when printing patterned structures. The current characteristics under different jet modes were studied, especially for the feature of peak current at the turning point of the trajectory. Based on the EDW current characteristics, a *CV* of 0.5 and a ratio of *I*_max_ to *I*_min_ of 7 were taken as boundary values to identify different jet ejection modes. The Taylor cone ejection mode had the smallest coefficient of variation of peak current, and it was the most stable mode for the accurate printing of micro/nano structures. Jet recognition based on the EDW micro/nano current provides a way of designing a control algorithm and realizing a closed-loop control system, which will promote the application of EDW technology.

## Figures and Tables

**Table 1:** Current characteristics in different jet modes.

Jet Mode	*I*_min_ (nA)	*I*_max_ (nA)	*I*_mean_ (nA)	*I*_max_/*I*_min_	*CV*
Droplet ejection mode	282.22 ± 42.88	1399.75 ± 161.52	684.54 ± 84.66	4.95 ± 0.44	0.83 ± 0.27
Taylor cone ejection mode	310.04 ± 40.36	1612.86 ± 107.95	828.01 ± 63.22	5.20 ± 0.48	0.32 ± 0.08
Retractive ejection mode	357.96 ± 72.65	3704.45 ± 122.02	1608.13 ± 529.59	10.34 ± 1.84	0.85 ± 0.23
